# Foot Health in People with Cancer Undergoing Chemotherapy: A Scoping Review

**DOI:** 10.3390/healthcare11111588

**Published:** 2023-05-29

**Authors:** Raquel Veiga-Seijo, Cristina Gonzalez-Martin

**Affiliations:** 1 Department of Health Sciences, Faculty of Nursing and Podiatry, Campus Esteiro, Campus Industrial de Ferrol, Universidade da Coruña, 15471 Ferrol, Spain; cristina.gmartin@udc.es; 2 Research Group in Nursing and Health Care, Instituto de Investigación Biomédica de A Coruña (INIBIC), Hospital Universitario de A Coruña (HUAC), Universidade da Coruña, Sergas, 15006 A Coruña, Spain; 3 Research Group in Rheumatology and Health (GIR-S), Faculty of Physiotherapy, Campus Oza, Universidade da Coruña, 15008 A Coruña, Spain

**Keywords:** cancer, foot, quality of life, chemotherapy, scoping review, evidence-based practice, oncology, podiatry

## Abstract

Background: Chemotherapy has relevant implications for cancer patients’ physical, social, and psychological health. Foot health has gained relevance in recent years due to its importance to independence and wellbeing, especially in chronic conditions. This study aims to explore the scope of the literature regarding foot health problems in people with cancer undergoing chemotherapy. Methods: scoping review following the PRISMA-ScR, Arksey and O’Malley, and the Joanna Briggs Institute guidelines. Different databases were used (Cochrane Plus, Scopus, Web of Science, and Pubmed). A total of 4911 articles were identified. Finally, 11 papers were included. Results: Foot problems are relevant and deteriorate wellbeing. The prevalence of some podiatric pathologies is controversial. The main literature deals with hand–foot syndrome and peripheral neuropathy. Focused instruments on foot health were not thoroughly used. Conclusion: There is insufficient evidence on foot health problems and their influence on the quality of life of people with cancer undergoing chemotherapy. Even though a significant percentage of this population has a foot problem, its care and importance are neglected. More studies are needed to contribute to the care of people with cancer through foot health.

## 1. Introduction

Cancer is a health condition with outstanding morbidity and mortality and is the second cause of death worldwide [1,2]. It presents an increasing trend worldwide, with an estimated 28.4 million cases in 2040 [3,4]. The relevance of this problem is shown in its magnitude and the implications that this health condition adds to the multidimensional complex of people’s wellbeing.

The quality of life (QoL) of people with cancer is one of the most relevant concerns in the field of oncology [5] since cancer is the disease that causes the most significant loss of years of healthy life [6]. This is due to the consequences that adverse treatment events have on people’s health, not only during treatment but also after it. Chemotherapy is one of the most common methods, and there is an increase in morbidity and mortality with the number of people requiring it [7]. Therefore, the development of adverse events seems to be an essential factor that contributes to the decrease in people’s QoL [8,9].

Several studies were found in the literature on the adverse effects that chemotherapy treatments trigger. Specifically, research over the past two decades has highlighted adverse effects, such as skin and nail toxicity or peripheral neuropathy [8,9], and their implications for people’s QoL. However, knowledge about these effects on foot health is invisible and neglected, despite the critical role that feet play in maintaining a healthy lifestyle [10,11]. In fact, only two articles refer to foot health [12,13].

At present, this gap in the literature is striking since other studies have shown the negative impact that podiatric problems have on general and health-related QoL, constituting a determinant of health [14]. In the field of oncology, this can also be very important, since people may feel limited in the way they face the disease process, in activities of daily living, in maintaining healthy lifestyle habits, and in having an active lifestyle within the possibilities during the oncological process.

In addition, the context described above is significantly different from the attention that other conditions have received in the scientific literature and in clinical practice in the field of foot health. In other health fields associated with chronic conditions [15], such as Diabetes Mellitus or Rheumatoid Arthritis, literature and implications for clinical practice are emerging in which foot problems are highly relevant, such as foot pain, structural alterations, and skin changes [16,17,18]. Likewise, today, the concern that amputations have for people with diabetes and health services is well known.

Taking into account the above context, the relevance and need to focus on foot health in this field are due to its role in posture and ambulation and its responsibility for autonomy, independence, and wellbeing [10]. In general, foot problems are known in the general population, and their identification is key since they have an impact on people’s general health. People with cancer have complex and multidimensional circumstances, and the current agenda requires interdisciplinary healthcare professionals to contribute to their QoL. Given that podiatric adverse events may interfere with the general and podiatric wellbeing of people with cancer, it is pertinent to better understand the effects on foot health in people receiving chemotherapy.

Up to now, no review has been found that explores the published literature on foot health problems in people with cancer undergoing chemotherapy. Therefore, it is necessary to determine to what extent and how this issue has been described in the literature. This scoping review aims to explore the scope of the literature regarding foot health problems in people with cancer undergoing chemotherapy.

For this reason, this study is proposed to summarize the current scientific evidence on the topic addressed, to determine the relevance of future research and in which direction it should be oriented, as well as to provide new information for clinical practice.

## 2. Materials and Methods

A systematic review was carried out based on the “Scoping Review” methodology, following the guidelines of Arksey and O’Malley [19] and PRISMA-ScR [20]. The purpose is to be an initial point of investigation in the exposed field, evaluating the magnitude and scope of the existing literature. Levac et al. [21] and guidelines from the Joanna Brigss Institute [22] were followed to ensure methodological rigor. According to its theoretical framework, this scoping review includes five phases that are shown below.

### 2.1. Identifying the Research Question (Stage 1)

The research question formulated in this scoping review is as follows: What is known about the foot health problems of people with cancer undergoing chemotherapy?

### 2.2. Identifying Relevant Studies (Stage 2)

A search strategy was carried out that involved several phases, beginning with the identification of key search terms. For this purpose, the Participant, Concept, and Context (PCC) framework recommended by the Joanna Briggs Institute [22] was taken into account: P: people with cancer older than 18 years, C: foot health problems, and C: chemotherapy treatment for cancer. From this framework, a test search was carried out with an initial strategy that was consulted and supported by specialists in database formation from the university. The final search strategy was carried out in February 2022 and reviewed in December 2022. Table 1 shows the search terms.

In accordance with the referenced theoretical framework [19], to identify potentially relevant documents, different electronic databases related to Health Sciences were used: Cochrane Library Plus, Pubmed, Web of Science, and Scopus. All original articles, reviews, and conference papers published in Spanish, Portuguese, and English in the last 15 years were considered for inclusion. Search terms were applied to the title, abstract, and keyword fields. Only those publications that addressed foot health problems in adult people receiving chemotherapy were included.

### 2.3. Study Selection (Stage 3)

All the authors reviewed the publications obtained and discussed the results. First, the documents were identified according to the title and abstract. Most of the discarded articles addressed the effectiveness of cancer therapies and were partly analyzed based on the adverse effects they triggered. Among them, reference was made to hand–foot syndrome, but without addressing foot health or responding to the objective of this work. Another significant volume of discarded papers addressed pathophysiological aspects of chemotherapy-induced peripheral neuropathy. Subsequently, the full text was reviewed, resolving disagreements if they occurred.

### 2.4. Charting the Data (Stage 4)

At first, the documents were saved in Mendeley and arranged in a Microsoft Word table. Two researchers reviewed each article. They were organized by author, year and place of publication, study population, methodology, and main results.

### 2.5. Collecting, Summarizing, and Reporting the Results (Stage 5)

The analysis was performed using a descriptive analysis (including *n* and percentage) and a thematic analysis (Braun and Clarke [23]), following both inductive and deductive approaches.

## 3. Results

**Figure 1:**
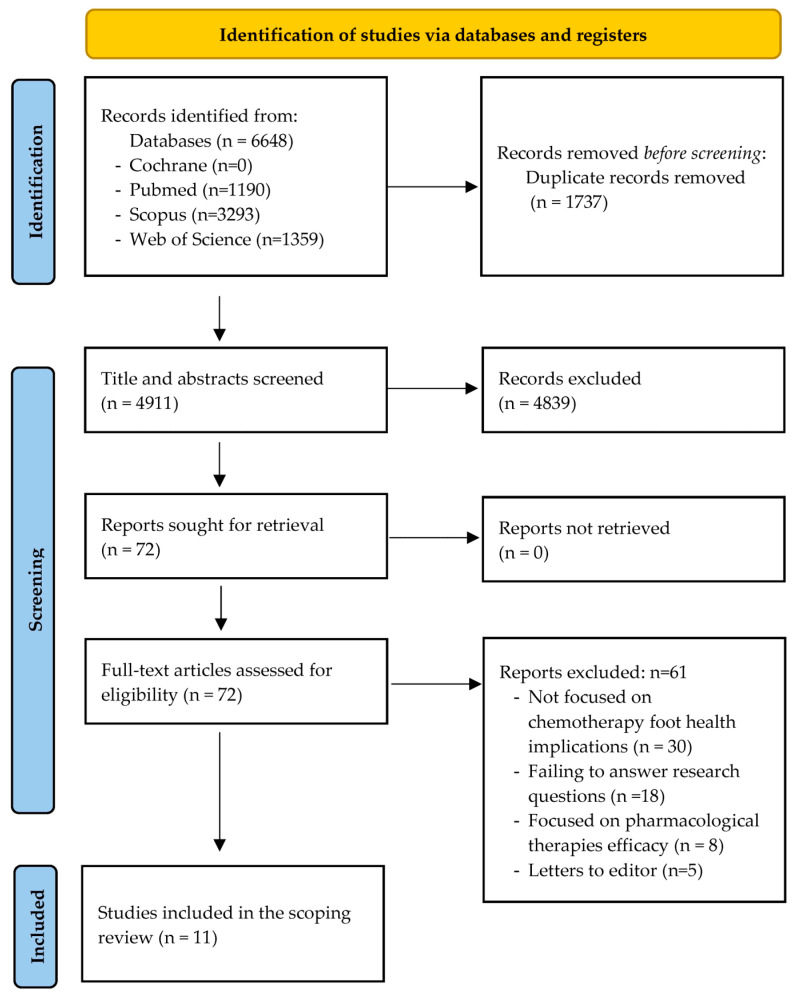
PRISMA flow diagram showing the studies included and excluded.

A total of 4911 articles were initially found. Finally, 11 articles fulfilled the previously established criteria (Figure 1). Table 2 shows the bibliometric and general characteristics of the studies analyzed, the foot health problems, and the foot health problems related to QoL in people with cancer undergoing chemotherapy.

### 3.1. Bibliometric Characteristics

Most of the included studies were published in 2019 (*n* = 3) [24,25,26], 2018 (*n* = 2) [13,27], and 2017 (*n* = 2) [12,28]. The oldest articles are from 2007 [29] y 2009 [30]. Four papers were published in Asia [24,25,26,27], three in Europe [12,29,31], three in North America [13,28,32], and one in Australia [30].

The interdisciplinary contributions to the subject come mainly from the departments of oncology (*n* = 4) [13,28,29,31], nursing (*n* = 3) [12,24,25], pharmacy (*n* = 3) [26,30,31], and dermatology (*n* = 3) [26,27,32]. Only two articles involved professionals specializing in foot health or podiatry [12,13].

The most studied type of cancer was breast cancer. Most of the articles studied taxane-based chemotherapy. Mainly, the documents included are quantitative observational studies (*n* = 5) and reviews (*n* = 3). Only one work is a qualitative study.

### 3.2. Methods of Collecting Information about the Foot

Six papers used the National Cancer Institute’s Common Terminology Criteria for Adverse Events. Two articles used scales related to hand–foot syndrome and QoL. The European Organization Peripheral Neuropathy Related QoL Scale was used in two investigations. Only one article used a specific questionnaire related to foot health and QoL (the Foot Health Status Questionnaire (FHSQ)), and another paper used in-depth interviews.

### 3.3. Main Objectives of the Documents Concerning the Foot

**Figure 2:**
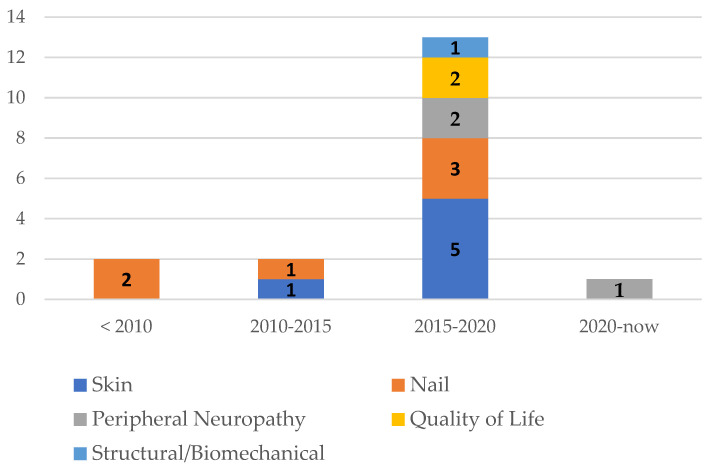
Main objectives of the documents concerning the foot/year of publication.

No study provided data on structural problems. Only one article addressed biomechanical problems (gait and balance) (Figure 2).

### 3.4. Findings from Thematic Analysis

The emerging themes of the analysis developed are shown below and in Table 2, which are not independent but are related to each other.

#### 3.4.1. Foot Health Adverse Events

A significant number of the studies focused on nail and skin toxicities [12,13,26,27,29,30,32]. Biswal and Mehta [27] showed the association between toxicities and the type of chemotherapy and reported that: 2.6% presented hand–foot syndrome associated with docetaxel and antibodies; 2.9% melanonychia, associated with cisplatin and paclitaxel; and 4.4% xerosis, usually manifested with 5-Fluorouracil. Miller et al. [32] reported that onycholysis arises from insults to the nail bed, is associated with taxanes, and has a prevalence of 0% to 44%. Beau’s lines, subungual hemorrhage, nail pigmentation, paronychia, and splinter hemorrhages are also associated with this drug (up to 88% of subjects). The remainder of the publications focused solely on hand–foot syndrome [25,32], which was associated in 89% of cases with doxorubicin and 5-Fluorouracil [32].

Montfort et al. [28] published the only study that addressed gait in peripheral neuropathy. They observed a decrease in balance (*p* = 0.001) and speed (*p* = 0.003). Lacouture et al. [13] and López-Palomo et al. [12] were the only ones to directly describe foot health problems (Table 2).

#### 3.4.2. Foot Health: A Critical Role Related to QoL of People Undergoing Anticancer Therapies

By focusing on foot health problems and using the analytic approaches mentioned above, the authors found that foot health problems related to QoL were highly highlighted in the included scientific literature. Consequently, this was a variable that emerged from the analysis of this study.

Thus, Palomo-López [12] studied podiatric adverse effects related to QoL among healthy women and those with breast cancer. Using the FHSQ, they observed significant differences in the domains of foot pain (*p* = 0.003), foot function (*p* < 0.001), physical activity (*p* < 0.001), social ability (*p* < 0.001), and vigor (*p* < 0.001)). Lacouture et al. [13] indicate that foot pain was scored at ≥8, and after the podiatry intervention, it improved to 4.8 ± 3.0 (*p* < 0.001). Similar results were obtained for QoL. Another study found that poorer QoL was also associated with decreased balance and walking speed (*p* < 0.05) [28].

Nail problems are associated with cosmetic problems, discomfort, pain, and impairment of QoL and activities of daily living [29,30,31], and can add morbidity and functional impairment [31]. On the other hand, Urakawa et al. [26] showed that the hand–foot syndrome had the highest statistically significant association with worse QoL (*p* < 0.05) among all the cutaneous adverse effects. Hsu et al. [25] observed that worse scores were obtained as the severity of toxicity increased (*p* < 0.05). In a qualitative study [24], participants identified this effect as a feeling of helplessness due to persistent symptoms. In addition, a body image problem arises since the appearance of the skin presents a dark coloration with a dirty appearance that they want to hide.

## 4. Discussion

The scientific evidence focused on foot health has grown significantly in the last 20 years, and the need for systematic reviews to develop evidence-based foot health science has been previously established [33]. The implications of foot health on QoL have been studied in other groups recently [34]. However, although cancer is a topic of great importance today and there is a lot of literature that addresses this health problem and the adverse effects of its therapies, there is a gap in the literature on its implications for foot health. The works found studied adverse effects in a general way or focused specifically and independently on any of them without focusing on foot health as a fundamental and determinant part of health [14].

This scoping review was conducted to illuminate to what extent foot health problems in people with cancer undergoing chemotherapy have been described in the literature. To the best of our knowledge, this is the first comprehensive review that attempts to address this issue. Only two articles addressed foot health, which were precisely the studies involving foot health professionals [12,13]. The information they provide suggests that their holistic study is essential since different organs are involved and have emotional, social, and physical consequences [34].

The lack of sufficient evidence did not allow us to compare information between studies nor to conclude to what extent and how it can be summarized in terms of foot problems related to QoL, this being the epicenter of future research. The description of foot health problems related to QoL in people with cancer undergoing chemotherapy is a theme and a variable that emerged from the first analysis of this scoping review. Foot health problems related to QoL were very prominent in all the papers articulated in the results, which allows us to have a more complete picture of this study area. Thus, QoL gained relevance, which is why it was included in the Results and Discussion sections. Overall, there has been a perceived gap in the literature since the development of the search strategy for this review. It is striking that the addition of the word “podiatry” did not yield results, which justifies the need to develop this area. Furthermore, it was surprising that the first articles were published in 2002, even though chemotherapy has been used for 80 years. Likewise, no research has been published in South America or Africa, despite the fact that the cancer data in these countries are overwhelming (11% increase in Africa and 101% increase in South America) [3].

It should be added that most of the public information that can be easily accessed is found in institutions such as the *National Cancer Institute*, the *American Cancer Society*, the *Spanish Society of Medical Oncology*, the *Canadian Cancer Society*, the *American Society of Clinical Oncology*, *Memorial Sloan Kettering Cancer Center*, the *Centers for Disease Control* and *Prevention, and Cancer Research UK*. These sources describe the adverse effects of cancer therapies. Only the National Cancer Institute includes information on skin and nail changes, although, like the reviewed literature, they only refer to the foot when describing hand–foot syndrome.

### 4.1. Literature Gap from the Foot Health Perspective: A Challenge for Discussion

Overall, the results showed that foot problems are relevant and impair wellbeing. Focused instruments on foot health were not thoroughly used. Mainly, scientific evidence addresses nail and skin problems. Initially, many articles were found, since the hand–foot syndrome is mentioned in many studies that address the adverse effects of treatments or their effectiveness [35]. However, they did not respond to the proposed objective. The oldest publications dealt with nail conditions. Since 2015, the evidence has covered a greater variety of topics (Figure 2). There was great disagreement about the magnitude of nail problems: while Gilbar et al. [30] mention that it is a practically unknown problem, Winther et al. [29] indicate that 88.5% present nail problems after three cycles of chemotherapy. Likewise, Zawar et al. [36] report that the most frequent nail changes were color change (54.26%) and nail dystrophy (29.45%), citing them as common effects. Instead, Miller et al. [32] reported other problems, such as onycholysis, with data ranging from 0% to 44%. They all agree that these conditions have received little attention in the oncology community. Furthermore, they indicate that much of the literature consists of case studies published in dermatology journals [30].

Regarding skin problems, 90% of people experience them at some point during treatment [37]. Hand-foot syndrome was very prominent in the results of this review [24,25], which is consistent with published evidence [38]. It is indicated that this condition had the highest degree of toxicity after the hematological one (25% of the cases). Alizadeh et al. [38] indicate that it occurs mainly between the third and sixth cycles of chemotherapy, which is consistent with the results presented. In addition, it is defined as a relevant cause for the suspension or limitation of treatment [39,40,41].

On the other hand, there is wide-ranging literature on the pathophysiological mechanisms of peripheral neuropathy. Although the foot is the most affected part, there is no literature that specifically focuses on what happens in the lower extremity [42]. By contrast, there is research on its involvement in QoL, gait, and balance [28]. Furthermore, a study [31] focused on QoL used scales that involved items related to the foot, which were the most prominent and negatively associated with QoL. It is noteworthy that no research has alluded to diabetes when the evidence points to it as a risk factor for peripheral neuropathy [43].

Lastly, Palomo-López [12] compared a sample of healthy women with breast cancer and chemotherapy, and Lacouture et al. [13] reported the clinical experience of the Oncology Department of a hospital in the Netherlands. They constitute the only works that revealed the implications of chemotherapy in the foot globally. They report problems such as hyperkeratosis (4%), xerosis (20%), digital deformities (7%), and paresthesias (19%), data that attract attention due to their low proportion. These studies focused on foot health report a greater diversity of effects, unlike the rest of the works included, which mostly only mentioned hand–foot syndrome or neuropathy. This agrees with two reports published in the United Kingdom. They describe some podiatric adverse effects that have been poorly described from a foot health perspective [44,45].

### 4.2. QoL of People with Cancer: An Agenda That Should Consider Foot Health

Cancer constitutes a threat to the wellbeing and perception of an individual’s QoL [46]. The increase in cancer survivors and the toxic effects of treatment that emerge during and after treatment have placed QoL in the critical focus of attention [47]. Although most toxicities are not the cause of a person’s death, they are critical to receiving treatment, enjoying adequate QoL during and after the disease process, and avoiding the loss of years of healthy life [31].

Even though no research has been found that addresses the implications that podiatric adverse effects have on QoL in a specific way, most of the included studies alluded to the negative contribution of these problems to people’s wellbeing and health. Only one article used a questionnaire that focused on foot health related to the person’s QoL [12] and not on each individual symptom. Therefore, we cannot compare this information with other studies, and we cannot conclude to what extent and how people’s QoL is influenced by podiatric adverse effects.

Winther et al. [29] found that 28.6% had mild, 8.6% moderate, and 5.7% severe functional problems due to nail changes. It affects one cosmetically and functionally and leads to a decrease in QoL. Although the data were not consistent between the included studies, they agreed that these problems were debilitating [38]. Regarding hand–foot syndrome, the included papers [24,25,26] mainly addressed its implications for QoL and daily activities. In the literature, a specific questionnaire was even found that assessed this aspect [48]. This effect stands out especially because it can severely present itself and lead to the suspension of chemotherapy, as shown by some case studies [49]. For this reason, new theories are emerging regarding this adverse effect, including the need for prevention, early detection, and daily care to maintain QoL and avoid further implications in the disease process, such as dose modification [26]. In addition, it is a prominent factor because it contributes to physical inactivity and falls [49].

Ganz and Dougherty [47] reflect on the importance of toxicities that emerge during treatment and after its completion, highlighting peripheral neuropathy due to its difficulty in managing even today. This agrees with other investigations [7], which report a correlation between adverse effects such as neuropathy and edema and a decrease in QoL. Other recent research shows that foot assessment methods are inconsistent among published studies, that the effects of neuropathy on foot health are most prominent, and that it adds morbidity to the cancer population [42]. Like hand–foot syndrome, this effect is associated with falls and balance problems [49]. Other literature states that neuropathy is associated with anxiety, depression, insomnia, and falls (*p* < 0.05) [50,51]. Finally, a point to note is that after treatment, problems such as neuropathy can be persistent and favor barriers related to sensorimotor and mobility deficits. This is associated with the possibility of not performing physical activity, decreased ambulation, and an increased body mass index, which may be correlated with other health problems [28,51].

### 4.3. Future Lines of Research and Clinical Practice

Future research should deepen knowledge about the influence of podiatric adverse effects on QoL in people with cancer undergoing chemotherapy. It is necessary to articulate research in other tumors, both sexes and other geographical locations. The current dearth of scientific evidence is linked to the fact that these effects are neglected in current oncological clinical practice. Only institutions and societies make indirect reference to these effects, but without focusing on the foot. This invites us to focus on this agenda from a comprehensive and holistic perspective, advancing research while providing guidelines through clinical practice guidelines. In addition, the fact that only the articles focused on foot health were carried out by healthcare professionals such as podiatrists and that the inclusion of the word “podiatry” in the search strategy did not produce any results justifies the need for these professionals to form part of the oncology work team.

What has been described above is related to the need to contribute to the current focus of attention for people with cancer: QoL. For that purpose, it is necessary to respond to a part of the knowledge that has not been described up to now. The need to: (a) develop standardized methods and specialized professionals for the assessment of the foot is evident, since their absence can hide the presence of podiatric problems; and (b) get involved in the symptom and attend to it through standardized guidelines [52]. In this sense, in addition to education on how to care for the foot and control symptoms, it is necessary to educate people about the adverse effects of anticancer drugs that people can develop, which will help with psychological trauma and improve QoL [27].

Particularly, hand–foot syndrome is the only dermal toxicity that does not present prevention or current treatment guidelines, despite being the most prevalent side effect, and that is related to a greater impact on QoL and dose limitation. Knowing that managing these effects can minimize treatment interruptions and improve people’s wellbeing, future research should focus on this aspect [53,54].

Clinical practice should be directed towards the prevention, diagnosis, and management of podiatric adverse events as an essential part of foot function and health-related quality of life and as an integral part of care, critical aspects for the care of people with cancer and survivors [13,26,40].

Consistent with what was observed in this study, in 2017, Lacouture et al. [13] reported that no podiatric screening and treatment have been developed to prevent or mitigate podiatric adverse events in cancer patients. As they described, a foot care program must include topics such as QoL and risk factors. Concerning peripheral neuropathy, recent research reports on the main complementary therapies to prevent or treat this side effect. It stands out in the exercise [42]. Concerning nail and dermal toxicities, such as paronychia or hand–foot syndrome, topical antibiotics, or urea cream [13].

Overall, the achievement of these improvements in practice must be based on person-centered care and a biopsychosocial approach. It is necessary that professionals, such as nurses and podiatrists, work together based on multidimensional interventions and person-centered self-care [55]. Nurses are in close contact with people, so the guidance provided during care will be essential. For this, it is necessary to listen to the demands and complaints of people in order to develop sensitive prevention and treatment strategies that are appropriate to their needs [47].

Therefore, the application of what is described in this review will guide not only professionals specialized in foot health but also oncologists, nurses, and especially the people themselves and their relatives/caregivers.

## 5. Conclusions

This research is the first to focus on foot health as a fundamental part of the quality of life of people with cancer undergoing chemotherapy. So far, no study has focused specifically on a sample of people with cancer receiving chemotherapy, and there is not sufficient information on how QoL is influenced by these adverse effects, which should be the epicenter of future investigations.

This study provides new insight into foot health, whose problems are diverse and relevant. Specifically, the problems described seem to favor people’s disabilities and affect activities of daily living. In addition, they imply the possibility of limiting or suspending the treatment dose, which highlights the problem analyzed. More studies are needed to contribute to the care of people with cancer through foot health.

## Figures and Tables

**Table 1:** Search terms.

Concept	Search Terms
Foot	Foot OR podiatry OR “hand–foot syndrome” OR “foot diseases” OR “foot health”
Cancer	Neoplasm OR cancer
Chemotherapy	“Drug therapy” OR “chemotherapy”

**Table 2:** Bibliometric characteristics, foot health problems, and foot health problems related to QoL in people with cancer undergoing chemotherapy [12,13,24,25,26,27,28,29,30,31,32].

First Author, Year, Country (Health Professional Department)	Journal	Sample	Anticancer Therapies	Type of Study	Research Objectives	Data Collection Methods Concerning Foot	Was the Aim of the Study the Foot Health?	Studied Foot Health Problems Related QoL	Main Findings
									*Foot health problems* *Foot health problems related to Quality of Life (QoL)*
Lacouture et al. [13], 2018, USA (Department of Oncology and Podiatry)	Journal of the American Podiatric Medical Association	*n* = 291 Breast (40.2%), colon (10.3%)	Cytotoxic chemotherapy, targeted therapy, radiation therapy, surgical procedures, and stem cell transplants	Clinical experience and literature review	To show the impact of podiatric adverse events on QoL in an Oncology Foot Care Program and to review podiatric adverse events related to cancer treatments.	NCICTCAE	Yes	Yes	*Foot health problems* Podiatric adverse events from anticancer therapies are highlighted. The main problems are nail toxicities, hand–foot syndrome (HFS), edema, xerosis, hyperkeratosis, and neuropathy. *Foot health problems related to QoL* The adverse effects negatively impact people’s QoL. Hence, it is important to prevent and manage podiatric adverse events because these effects may result in the interruption, diminution, or discontinuation of cancer treatments.
Komatsu et al. [24], 2019, Japan, Yagasaki, Hirata, and Hamamoto (Department of Nursing)	European Journal of Oncology Nursing	*n* = 20 (13 female, 7 male) colon (65%), gastric (20%) cancer	Chemotherapy and targeted therapy	Qualitative study	To understand the perceived needs of advanced-stage cancer patients with chemotherapy-related hand–foot syndrome and/or targeted therapy-related hand–foot skin reactions.	Interviews	Focused on HFS	Yes	*Foot health problems* Participants experienced pain and a change in sensitivity, describing the feet as an integral part of mobility, which affects the activities of daily living. *Foot health problems related to QoL* Four themes emerged about the needs of people with chemotherapy-related HFS: a sense of helplessness with persistent symptoms, noticeable appearance as a barrier to social participation, decreased willingness to work and continue treatment, and the need for individual coping strategies. These unmet needs are not often voiced. Hence, health care providers must be involved in this problem.
Hsu et al. [25], 2019, Taiwan (Department of Nursing)	European Journal of Oncology Nursing	*n* = 85 breast cancer (100% female)	Docetaxel-based chemotherapy	Cross-sectional, descriptive, and correlational designs	(1) to assess breast cancer patients’ perceived levels of HFS-related foot symptoms, HFS-related hand or finger symptoms, and HFS-related restrictions in daily activities, and (2) to identify factors associated with HFS-related restrictions in daily activities.	Hand–Foot Quality of Life Scale (HF QoLS) symptom subscale questionnaire HF QoLS—daily activity subscale NCICTCAE	Focused on Hand–Foot Syndrome	Yes	*Foot health problems* The foot stands out as the body part most affected in relation to HFS. Participants reported a higher level of HFS-related foot symptoms than in hands or fingers. They observed that 41.2% of people with breast cancer reported this effect. The symptoms presented a higher mean in the feet (8.76 ± 0.72) than in the hands (7.62 ± 0.70). *Foot health problems related to QoL* The restriction in daily activities was associated more with foot symptoms, which explained 44.7% of the variance in the restriction of activities.
Miller et al. [32], 2014, USA (Department of Dermatology)	Journal of the American Academy of Dermatology	Breast cancer	Different chemotherapy agents and targeted multikinase inhibitors	Review	To describe the epidemiology, pathogenesis, clinical presentation, and current evidence-based treatment for chemotherapy-induced hand–foot syndrome and nail changes.	HFS: National Cancer Institute Criteria for its classification and World Health Organization criteria Nail: NCICTCAE	Focused on Hand–Foot Syndrome and nail changes (both hand and foot)	Yes	*Foot health problems* HFS can affect palms, soles, dorsal hands, and feet, with erythema and edema accompanied by the onset of neuropathic pain. It can progress to blistering with desquamation, erosion, and ulceration. HFS was initially described as tingling and burning pain. Subsequently, the sensation of pain and temperature decrease, and neuropathic symptoms begin, accompanied by erythema and edema palmoplantar. Nail changes such as onycholisis, Beau lines, subungual hemorrhage, nail pigmentation, paronychia, and splinter hemorrhage can occur in up to 88% of patients. *Foot health problems related to QoL* These problems may cause cosmetic concern, pain, infection, and an impact on QoL. They may involve reducing the dose of chemotherapy or even having to stop treatment and switch to another. They are common complications of many classic agents.
Gilbar et al. [30], 2009, Australia (Department of Pharmacology)	Journal of Oncology Pharmacy Practice	Articles about breast, lung, ovarian, and colon cancer, melanoma, Hodgkin, and carcinoid tumors	Different chemotherapy agents	Literature Review	To provide a comprehensive literature review of chemotherapy-induced nail toxicity (presentation, implication, drugs, and approaches for prevention and management).	NCICTCAE	Focused on nails (both hand and foot)	No	*Foot health problems* Nail toxicity is a relatively uncommon adverse effect. The clinical presentation varies depending on the chemotherapeutic agent, the nail structure affected, and the severity. Taxane and anthracyclines are the most prevalent drugs related to this toxicity. The most common nail problems were: Beau’s lines, onychomadesis, Mees’s lines, melanonychia, onycholysis, Muehrcke’s lines, splinter hemorrhage, subungual hematoma, and paronychia. *Foot health problems related to QoL* It may involve cosmetic concerns, pain, discomfort, and negative implications for QoL and DLA. It is necessary to develop a healthcare program about the potential nail toxicities.
Palomo-López et al. [12], 2017, Spain (Department of Nursing and Podiatry)	Cancer Management and Research	*n* = 200 100% women (50% breast cancer, 50% without cancer)	Chemotherapy treatment	Case-control observational	To analyze and compare foot health and general health in a sample of women with breast cancer and healthy women.	Foot Health Status Questionnaire and podiatric assessment	Yes	Yes	*Foot health problems* Women with breast cancer reported 94% of foot problems. The most frequent alterations in the feet were: nail abnormalities (46%), pain (36%), cracks and dryness (20%), paresthesia (19%), inflammation (10%), varices (8%), deformed fingers (7%), and helomas and hardness (4%). Other problems reported were cramps, loss of sensation, or blisters. *Foot health problems related to QoL* Women with breast cancer have a lower foot health-related QoL compared with healthy women. Clinical aspects, with an emphasis on foot pain and disability, were increased. Physical activity, social capacity, and vigor were affected. Therefore, more attention should be paid to the general health care and prevention of foot problems in breast cancer survivors.
Engvall et al. [31], 2022, Sweden (Department of Oncology and Pharmacology)	Breast Cancer Research and Treatment	646 survivors of breast cancer	Post-taxane treatment	Cross-sectional cohort study	To explore the impact of persistent sensory and motor taxane-induced peripheral neuropathy symptoms on health-related QoL among early-stage breast cancer survivors.	EORTC QLQ-C30, HADS, CIPN20	Focused on peripheral neuropathy	Yes	*Foot health problems related to QoL* TIPN has a significant impact on global QoL. The main important things were “difficulty walking because of foot drop” and “problems standing/walking because of difficulty feeling ground under feet”. The authors reveal that although it is not usually life-threatening, it can significantly affect QoL.
Winther et al. [29], 2007, Denmark (Department of Oncology)	Supportive Care Cancer	*n* = 55 breast cancer	Chemotherapy treatment (docetaxel)	Observational and prevalence study	To estimate the frequency and severity of nail changes due to treatment.	NCICTCAE	No (nails, both hands and feet)	Yes	*Foot health problems* A total of 88.5% reported changes in the nails after three cycles, 37.2% took place in the foot, 42.9% developed functional problems, and 37% had problems finding proper footwear. The association between nail changes and fungal infection was not found. They observed that the prevalence and aesthetic implications are similar in hands and feet, although the trend was higher in the hands. They found a statistically significant relationship between having neuropathy and nail changes in the feet (*p* < 0.001). *Foot health problems related to QoL* Nail changes have both cosmetic and functional impact, which may lead to a decrease in QoL. A total of 28.6% presented mild functional problems, 8.6% moderate problems, and 5.7% severe problems due to nail changes.
Monfort et al. [28], 2017, United States (Department of Oncology, biostatistics, engineering)	Breast Cancer Research and Treatment	*n* = 33 breast cancer patients (32 female/1 male)	Taxane	Longitudinal study	To describe symptoms of CIPN and functional impairments.	Standing balance, gait, Modified Total Neuropathy Score, Patient Report Outcomes (EORTC QLQ-CIPN20, EORTC QLQ-C30, BPI-SF)	No (peripheral neuropathy—gait)	Yes	*Foot health problems* Significant negative changes were observed concerning gait, balance, and symptoms reported by people. A worsening of sensory, motor, and autonomic symptoms and pain has been reported with cumulative taxane exposure. They observed that, in the first cycle of chemotherapy, the balance was reduced in 28% of cases and walking speed in 5%. *Foot health problems related to QoL* Worsened physical functioning was shown on the EORTC QLQ-C30.
Biswal et al. [27], 2018, India (Deparment of Dermatology)	Indian Journal of Dermatology	*n* = 1000 Different tumor (24.1% genitourinary, 14.7% breast)	Chemotherapy drugs in combination (mostly alkylating agents)	Observational study	To know the cutaneous adversities in patients undergoing chemotherapy and the drug(s) most commonly associated with them.	Clinical manifestations	No (cutaneous adversities)	No	*Foot health problems* Different cutaneous adversities of chemotherapy were found, such as xerosis (4.4%), melanonychia (2.9%), or HFS (2.6%). *Foot health problems related to QoL* It is pointed out that it is important to improve knowledge about adverse effects of anticancer drugs, which will help reduce psychological trauma and improve QoL.
Urakawa, et al. [26], 2019, Japan (Department of Dermatology and Pharmacology)	Journal of Cancer	*n* = 67 Cancer patients	Skin-toxic chemotherapeutic agents	Cross-sectional study	To investigate which skin toxicities influenced QoL and to what extent.	NCICTCAE, Dermatology Life Quality Index, and Skindex	No (skin toxicities)	Yes	*Foot health problems* This research studied general skin toxicities. A total of 21 subjects developed paronychia, and 25 developed hand–foot syndrome. *Foot health problems related to QoL* HFS was a stronger factor in decreasing QOL than xerosis, paronychia, pigmentation, or rash. Therefore, especially in HFS, prevention, early detection, and daily medical care are necessary to maintain QOL.

BPI-SF: Brief Pain Inventory-Short Form; CIPN: Chemotherapy-Induced Peripheral Neuropathy; EORTC QLQ-CIPN 20: European Organization for Research and Treatment of Cancer Quality of Life Questionnaire-Chemotherapy-Induced Peripheral Neuropathy; EORTC QLQ-C30: European Organization for Research and Treatment of Cancer Quality of Life Questionnaire; DLA: Daily Living Activities; HADS: Hospital Anxiety and Depression Scale; HF QoLS: Hand–Foot Quality of Life Scale; HFS: Hand–Foot syndrome; NCICTCAE: National Cancer Institute Common Terminology Criteria for Adverse Events; QoL: quality of life; TIPN: Taxane-Induced Peripheral Neuropathy.

## Data Availability

Not applicable.
